# Antibodies, B Cell Responses and Immune Responses to SARS-CoV-2 Infections

**DOI:** 10.3390/antib12010012

**Published:** 2023-02-01

**Authors:** Luis Martinez-Sobrido, James J. Kobie

**Affiliations:** 1 Texas Biomedical Research Institute, San Antonio, TX 78227, USA; 2 Department of Medicine, Division of Infectious Diseases, University of Alabama at Birmingham, Birmingham, AL 35294, USA

Coronaviruses (CoV) are enveloped, positive-sense, single-stranded RNA viruses responsible for causing seasonal, mild respiratory disease in humans [1,2]. They include the endemic human CoVs NL63, 229E, OC43, and HKU1, which are associated with mild respiratory illnesses [1,2]. However, three CoVs have been responsible for significant morbidity and mortality in humans: Severe Acute Respiratory Syndrome CoV (SARS-CoV), which spread in 2003 [3,4], Middle East Respiratory Syndrome CoV (MERS-CoV), which appeared in 2012 [3,4], and Severe Acute Respiratory Syndrome CoV-2 (SARS-CoV-2), which is responsible for the ongoing coronavirus disease 2019 (COVID-19) pandemic [5,6,7,8].

SARS-CoV-2 emerged in the city of Wuhan, China, at the end of 2019 and has dramatically impacted public health and socioeconomic activities around the world [9,10], mainly due to its high transmissibility [11,12]. The explosive emergence of SARS-CoV-2 infection in humans has resulted in alarming case fatalities, rivaling the “Spanish flu” pandemic of 1918. As of November 2022, SARS-CoV-2 has been responsible for over 645 million infections and more than 6.6 million human deaths (https://COVID-19.who.int (accessed on 28 November 2022)). Several prophylactic-subunit-, inactivated, mRNA-, and vector-based vaccines and therapeutic antivirals or monoclonal antibodies have been developed for SARS-CoV-2. To date, the United States Food and Drug Administration (FDA) has authorized the use of three types of vaccines in humans: Spikevax (formerly Moderna), COMIRNATY (formerly BioNTech and Pfizer), and Janssen [13,14]; four antiviral medications: Remdesivir, baricitinib, molnupiravir, and nirmatrelvir; and one monoclonal antibody (MAb): bamlanivimab [15,16,17], for the treatment of SARS-CoV-2 infection. Unfortunately, SARS-CoV-2 has rapidly accumulated mutations, leading to the emergence of variants of concern (VoC) and variants of interest (VoI), jeopardizing the effectiveness of existing preventive and/or treatment options [18,19,20,21,22].

Since the emergence of SARS-CoV-2, significant advances have been made in understanding the biology of the virus, developing vaccines, and identifying effective antivirals or neutralizing antibodies. This Special Issue, “Antibodies, B Cell Responses and Immune Responses to SARS-CoV-2 Infections”, assembles a collection of six new research articles, three reviews, one case report, and one perspective document, which cover vaccine immunogenicity and protection efficacy, classical and new antigen targeting, conserved viral antigens and epitopes, the identification and characterization of SARS-CoV-2 cross-reactive and broadly neutralizing antibodies, the induction of efficient and protective adaptive B cell responses, and the correlation of B cell activation and induction of antibody protection.

The first article, “Kinetics of the neutralizing and spike SARS-CoV-2 antibodies following the Sinovac inactivated virus vaccine compared to the Pfizer mRNA vaccine in Singapore”, compares the kinetics of total and neutralizing SARS-CoV-2 antibodies to three doses of the Sinovac inactivated virus vaccine and the Pfizer mRNA-based vaccine from January 2021 to February 2022 in Singapore [23]. The authors found that the Pfizer mRNA-based vaccine was able to generate more robust total and neutralizing antibody responses than those induced by vaccination with the inactivated Sinovac vaccine after a first, second, and third vaccination [23]. The second manuscript, “MALDI-TOF-MS-based identification of monoclonal murine anti-SARS-CoV-2 antibodies within one hour”, demonstrates how previously described MALDI-TOF-MS fingerprinting [24] can be used to identify, within minutes, monoclonal antibodies and their subclasses against SARS-CoV-2 [25]. In the next document, “Fc-independent protection from SARS-CoV-2 infection by recombinant human monoclonal antibodies”, the authors characterize two SARS-CoV-2-neutralizing human monoclonal antibodies, or hMAbs (MD65 and BLN1), targeting the receptor-binding domain (RBD) and the N-terminal domain (NTD), respectively, of the SARS-CoV-2 spike (S) glycoprotein, that were engineered to contain a Fc domain with three mutations (N297G, S298G, and T299A) that eliminates glycosylation and the binding to FcgR and to the complement system activator C1q. They show that both modified hMAbs were able to retain in vitro neutralization activity and protection efficacy, both prophylactically and therapeutically, in the K18 hACE2 transgenic mouse model, against lethal challenge with SARS-CoV-2, demonstrating the Fc-independent protection against SARS-CoV-2 of the two hMAbs [26]. In the manuscript “From anti-SARS-CoV-2 immune response to the cytokine storm via molecular mimicry”, Darja Kanduc determines the role of molecular mimicry in the cytokine storms induced by SARS-CoV-2 infection [27]. The study shows that SARS-CoV-2 S glycoprotein shares immune determinants with 53 anti-inflammatory human proteins, and that this molecular mimicry is responsible for SARS-CoV-2-induced cytokine storms observed during SARS-CoV-2 infection in COVID-19 patients [27]. In a separate manuscript, “From anti-SARS-coV-2 immune responses to COVID-19 via molecular mimicry”, Darja Kanduc also investigates the autoimmune potential of SARS-CoV-2 infection and identifies immunoreactive epitopes in SARS-CoV-2 that matches those present in human proteins, and how this mimicry could be responsible for some of the diseases associated with SARS-CoV-2 infection [28]. Finally, in “Viroinformatics-based analysis of SARS-CoV-2 core proteins for potential therapeutic targets”, the authors use bioinformatics-based drug-discovery approaches to identify effective antiviral drugs targeting structural viral proteins for the treatment of SARS-CoV-2 infection [29].

In the second section, comprising review articles, “Long-term immunity and antibody response challenges for developing efficient COVID-19 vaccines” addresses different questions and concerns related to the immunogenicity and protection efficacy of COVID-19 vaccines since the use of the BNT162b2 mRNA-based Pfizer vaccine in 2020, including the need for vaccine boosters due to a decline in antibody titers or the lack of cross-reactivity of vaccine-induced antibodies against newly identified VoCs [30]. In “Cellular, antibody and cytokine pathways in Children with acute SARS-CoV-2 and MIS-C: Can we match the puzzle”, Lazova et al. review the literature to investigate T-cell, antibody and cytokine responses, and other related conditions, such as multisystem inflammatory syndrome (MIS-C), in the disease outcome of SARS-CoV-2 infection [31]. The last review document, “Structural features and PF4 function that occur in heparin-induced thrombocytopenia (HIT) complicated by COVID-19”, provides a systematic review of the functions of platelets in infectious diseases, mainly in COVID-19 patients, to provide a better understanding on how platelet factor 4 (PF4) and the potential use of a PF4-blocking antibody could be used to manage heparin-induced thrombocytopenia (HIT) [32].

In the unique case report “The course of SARS-CoV-2 infection was not severe in a Crohn’s patient who administered maintenance anti-TNF therapy overlapping the early pre-symptomatic period of infection”, the authors present a case of a 60-year-old female with Crohn’s disease who was inadvertently administered with anti-cytokine therapy during the pre-symptomatic period of SARS-CoV-2 infection, and how, despite this immune suppression medication, the patient did not experience a more severe COVID-19 disease outcome [33].

Finally, the single perspective manuscript “Is the host viral response and the immunogenicity of vaccines altered by pregnancy?” provides literature evidence that pregnant women are more susceptible to respiratory viral infections and may not respond effectively to vaccines, making them more vulnerable to developing serious complications from respiratory viral infections. The work also elaborates on how new methods of measuring vaccine efficacy in this high-risk group could help to determine vaccine efficacy [34].

We hope that the manuscripts published in this Special Issue represent, to some extent, the most current advances related to research on antibodies and B cell and immune responses to SARS-CoV-2 infection and vaccination. We also hope that the articles in this Special Issue encourage other researchers to conduct future studies aiming to understand antibody and B cell responses to SARS-CoV-2, or other viral infections and/or vaccinations. Moreover, we hope the manuscripts in this Special Issue open the door to collaborations with the goal of improving the development of vaccines for the efficient control of SARS-CoV-2 infection and the ongoing COVID-19 pandemic.

Finally, we would like to thank all the authors that contributed to this Special Issue for their participation and for taking the time to submit manuscripts. Likewise, we want to also thank the Editorial Office at *Antibodies* for their help, guidance and assistance in putting together this Special Issue.
